# Detection of Merkel cell polyomavirus in cervical squamous cell carcinomas and adenocarcinomas from Japanese patients

**DOI:** 10.1186/1743-422X-9-154

**Published:** 2012-08-09

**Authors:** Masayuki Imajoh, Yumiko Hashida, Yuiko Nemoto, Hiroyoshi Oguri, Nagamasa Maeda, Mutsuo Furihata, Takao Fukaya, Masanori Daibata

**Affiliations:** 1 Department of Microbiology and Infection, Kochi Medical School, Kochi University, Nankoku, Kochi, 783-8505, Japan; 2 Department of Obstetrics and Gynecology, Kochi Medical School, Kochi University, Nankoku, Kochi, 783-8505, Japan; 3 Department of Pathology, Kochi Medical School, Kochi University, Nankoku, Kochi, 783-8505, Japan

**Keywords:** Merkel cell polyomavirus, Cervical squamous cell carcinoma, Cervical adenocarcinoma, HPV typing

## Abstract

**Background:**

Merkel cell polyomavirus (MCPyV) was identified originally in Merkel cell carcinoma (MCC), a rare form of human skin neuroendocrine carcinoma. Evidence of MCPyV existence in other forms of malignancy such as cutaneous squamous cell carcinomas (SCCs) is growing. Cervical cancers became the focus of our interest in searching for potentially MCPyV-related tumors because: (i) the major histological type of cervical cancer is the SCC; (ii) the uterine cervix is a common site of neuroendocrine carcinomas histologically similar to MCCs; and (iii) MCPyV might be transmitted during sexual interaction as demonstrated for human papillomavirus (HPV). In this study, we aimed to clarify the possible presence of MCPyV in cervical SCCs from Japanese patients. Cervical adenocarcinomas (ACs) were also studied.

**Results:**

Formalin-fixed paraffin-embedded tissue samples from 48 cervical SCCs and 16 cervical ACs were examined for the presence of the MCPyV genome by polymerase chain reaction (PCR) and sequencing analyses. PCR analysis revealed that 9/48 cervical SCCs (19%) and 4/16 cervical ACs (25%) were positive for MCPyV DNA. MCPyV-specific PCR products were sequenced to compare them with reference sequences. The nucleotide sequences in the MCPyV large T (*LT*)-sequenced region were the same among MCPyV-positive cervical SCCs and AC. Conversely, in the MCPyV viral protein 1 (*VP1*)-sequenced region, two cervical SCCs and three cervical ACs showed several nucleotide substitutions, of which three caused amino acid substitutions. These sequencing results suggested that three MCPyV variants of the VP1 were identified in our cases. Immunohistochemistry showed that the LT antigen was expressed in tumor cells in MCPyV-positive samples. Genotyping of human HPV in the MCPyV-positive samples revealed that infected HPVs were HPV types 16, 31 and 58 for SCCs and HPV types 16 and 18 for ACs.

**Conclusions:**

This study provides the first observation that MCPyV coexists in a subset of HPV-associated cervical cancers from Japanese patients. The prevalence of MCPyV in these lesions was close to that observed in the cutaneous SCCs. Further worldwide epidemiological surveys are warranted to determine the possible association of MCPyV with pathogenesis of cervical cancers.

## Background

Polyomaviruses are small, nonenveloped DNA viruses with icosahedral capsids containing a circular double-stranded DNA genome. Merkel cell polyomavirus (MCPyV) was discovered in Merkel cell carcinoma (MCC) [1]. MCC is a rare but aggressive neuroendocrine carcinoma of the skin, characterized by the common clinical features known as “AEIOU”: namely, asymptomatic, expanding rapidly, immunosuppressive, occurring in patients older than 50 years and located in ultraviolet-exposed sites [2]. Subsequent studies confirmed a more frequent prevalence of MCPyV in MCCs from North American and European patients than in Australian patients [3-15]. Among Asian patients, MCPyV has been shown to be present in 55–79% of MCCs in Japan [16-18].

The MCC tumor is probably derived from primitive epidermal stem cells [19], but its origin is still controversial. Conversely, its fine structure has been well studied and immunohistochemistry is important for diagnosing MCCs as they express both epithelial and neuroendocrine markers [20]. Among these, cytokeratin (CK) 20, a cytoskeleton of intermediate filaments, is the most specific and sensitive marker for detecting MCCs [21]. This immunophenotype has been reported for some cervical neuroendocrine carcinomas [22]. However, in that report, all CK20-positive cases showed a negative reaction with CM2B4, a monoclonal antibody against an antigenic epitope on the MCPyV large T (LT) antigen [23]. Since then, the association of MCPyV with cervical cancers has not been studied.

The route of MCPyV transmission has not been established, but MCPyV was detected in respiratory tract secretions [24-27]. If MCPyV is transmitted during sexual activity, it is conceivable that the uterine cervix would be exposed to this virus. In fact, MCPyV was also detected in the oral and anogenital mucosa of human immunodeficiency virus-positive individuals [28,29]. These findings prompted us to investigate the existence of MCPyV in cervical cancers.

The cutaneous squamous cell carcinoma (SCC) is the second most frequent skin cancer [30]. MCCs can arise as a population admixed with SCC cells in the same lesion [31-33]. MCPyV has also been detected in cutaneous SCCs at a lower frequency than MCCs [34-37]. Our previous study showed that MCPyV was present in 13% of cutaneous SCCs from Japanese patients [38].

The major histological type of cervical cancer is the SCC in which human papillomavirus (HPV) is recognized as a causative agent. The aims of this study were to investigate whether MCPyV exists in cervical SCCs, as seen in cutaneous SCCs from Japanese patients. Cervical adenocarcinomas (ACs) were also examined for the existence of MCPyV.

## Results

### Detection of MCPyV DNA

Formalin-fixed paraffin-embedded (FFPE) resection specimens from 48 Japanese cervical SCCs (denoted as cases SCC1 to SCC48) and 16 cervical ACs (cases AC1 to AC16) were screened for the presence of MCPyV DNA by polymerase chain reaction (PCR) analysis with two primer sets targeting the MCPyV *LT* and viral protein 1 (*VP1*) genes. The PCR results are summarized in Table 1. Of the 48 cervical SCCs, MCPyV DNA was detected in eight cases (SCC1, 3, 30, 31, 35, 36, 39 and 48) with the LTsh primers and in two cases (SCC3 and 47) with the VP1 primers. Case SCC3 was positive for MCPyV with both the VP1 and LTsh primers. Of 16 cervical ACs, MCPyV DNA was detected in one case (AC15) with the LTsh primers and in three cases (AC6, 7 and 16) with the VP1 primers. Overall, nine cervical SCCs (19%) and four cervical ACs (25%) were positive for MCPyV using either LTsh or VP1, or both primers. The β-globin gene was amplified consistently in all samples (data not shown). Clinical stages of the patients with SCCs harboring MCPyV DNA were as follows: stage 0, 4 patients; Ia, 3 patients; IIa, 1 patient; and IIb, 1 patient. The MCPyV genomes in patients with ACs were all detected from samples at clinical stage Ib. The MCPyV DNA-positive samples were all negative for CK20 and chromogranin by immunohistochemistry, indicating the absence of a neuroendocrine immunophenotype (Table 1).

**Table 1:** Summary of clinicopathological data and results of PCR, real-time PCR and immunohistochemistry analyses in MCPyV DNA-positive cases

**Case**	**Clinical information**			**PCR**			**Real-time PCR**	**Immunohistochemistry**		
	**Type**	**Age**	Stage^*^	**MCPyV LTsh**	**MCPyV VP1**	**HPV type**	**MCPyV DNA load (copies per cell)**	**MCPyV LT**^**†**^	**CK20**	**Chromogranin**
1	SCC	45	IIa	+	–	16	0.00014	–	–	–
3	SCC	74	IIb	+	+	16	0.0013	2+	–	–
30	SCC	29	0	+	–	16	0.00035	–	–	–
31	SCC	42	0	+	–	16	0.0021	–	–	–
35	SCC	59	Ia	+	–	58	0.00027	3+	–	–
36	SCC	38	0	+	–	16	0.00033	1+	–	–
39	SCC	33	0	+	–	58	0.00067	1+	–	–
47	SCC	38	Ia	–	+	31	0.00073	–	–	–
48	SCC	46	Ia	+	–	16	0.00081	2+	–	–
6	AC	50	Ib	–	+	18	0.00064	–	–	–
7	AC	61	Ib	–	+	16	0.00037	–	–	–
15	AC	54	Ib	+	–	18	0.00095	3+	–	–
16	AC	72	Ib	–	+	16	0.0015	–	–	–

^*^According to the FIGO clinical staging.

^†^Immunohistochemistry with CM2B4 monoclonal antibody.

### MCPyV DNA sequencing

**Figure 1:**
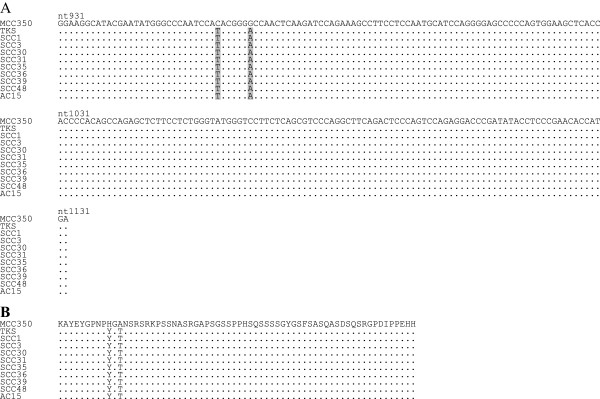
**Multiple nucleotide alignments (A) and amino acid alignments (B**) **of the *LT*-sequenced region in cases positive for MCPyV DNA with LTsh primers.** Nucleotide substitutions are shown with gray boxes: C → T at position 960 in TKS and all cases; and G → A at position 966 in TKS and all cases. The two nucleotide substitutions resulted in amino acid substitutions (H → Y and A → T, respectively). Nucleotide numbers refer to the sequence of MCC350 (GenBank accession number EU375803).

**Figure 2:**
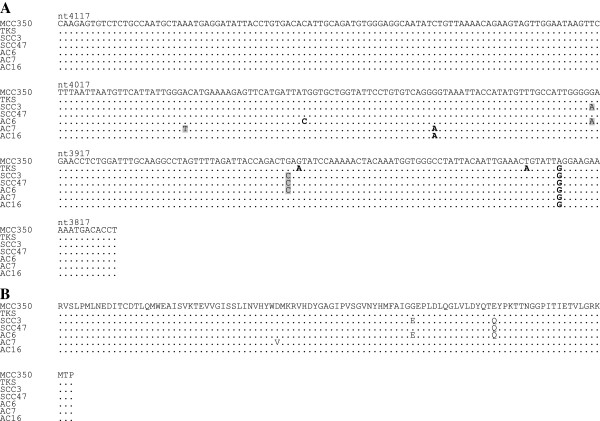
**Multiple nucleotide alignments (A) and amino acid alignments (B) of the *VP1*-sequenced region in cases positive for MCPyV DNA with VP1 primers.** Nucleotide substitutions resulting in amino acid substitutions are shown with gray boxes and the other nucleotide substitutions are shown in boldface: A → T at position 3994 in case AC7; T → C at position 3972 in case AC6; G → A at position 3948 in cases AC7 and AC16; G → A at position 3919 in cases SCC3 and AC6; G → C at position 3875 in cases SCC3, SCC47 and AC6; G → A at position 3873 in TKS; T → A at position 3831 in TKS; and A → G at position 3825 in TKS and all cases. Amino acid substitutions were D → V in case AC7, G → E in cases SCC3 and AC6, and E → Q in cases SCC3, SCC47 and AC6

The PCR-positive products were sequenced to confirm that they contained MCPyV-specific DNA and to compare their results with reference sequences of the MCC350 genome isolated from cases of North American MCC [1] and the TKS genome isolated from cases of Japanese Kaposi sarcoma [16]. Eight cervical SCCs and one cervical AC had identical nucleotide substitutions at positions 960 and 966 in the *LT*-sequenced region, followed by amino acid substitutions (Figure 1). The nucleotide sequences were also 100% homologous with that of TKS. Two cervical SCCs and three cervical ACs showed the following nucleotide substitutions in the *VP1*-sequenced region: T instead of A at position 3994 in case AC7; C instead of T at position 3972 in case AC6; A instead of G at position 3948 in cases AC7 and AC16; A instead of G at position 3919 in cases SCC3 and AC6; C instead of G at position 3875 in cases SCC3, SCC47 and AC6; and G instead of A at position 3825 in all cases (Figure 2A). Of these, the nucleotide substitutions at positions 3994, 3919, and 3875 caused amino acid substitutions (Figure 2B). Consequently, one amino acid substitution was present at different positions between cases SCC47 and AC7, whereas cases SCC3 and AC6 shared a common amino acid sequence. On the other hand, the nucleotide substitutions in case AC16 did not result in amino acid substitutions. Thus, three distinguishable variants of the VP1 were identified in our cases.

### Quantification of MCPyV DNA

MCPyV DNA loads were determined in the PCR-positive cases with either LTsh or VP1 primers. As shown in Table 1, nine SCCs contained MCPyV DNA sequences ranging from 0.00014 to 0.0021 copies per cell (median = 0.00067), while four ACs contained MCPyV DNA sequences ranging from 0.00037 to 0.0015 copies per cell (median = 0.0008). These DNA loads were lower than those in Japanese MCCs reported previously [17].

### Expression of the MCPyV LT antigen

**Figure 3:**
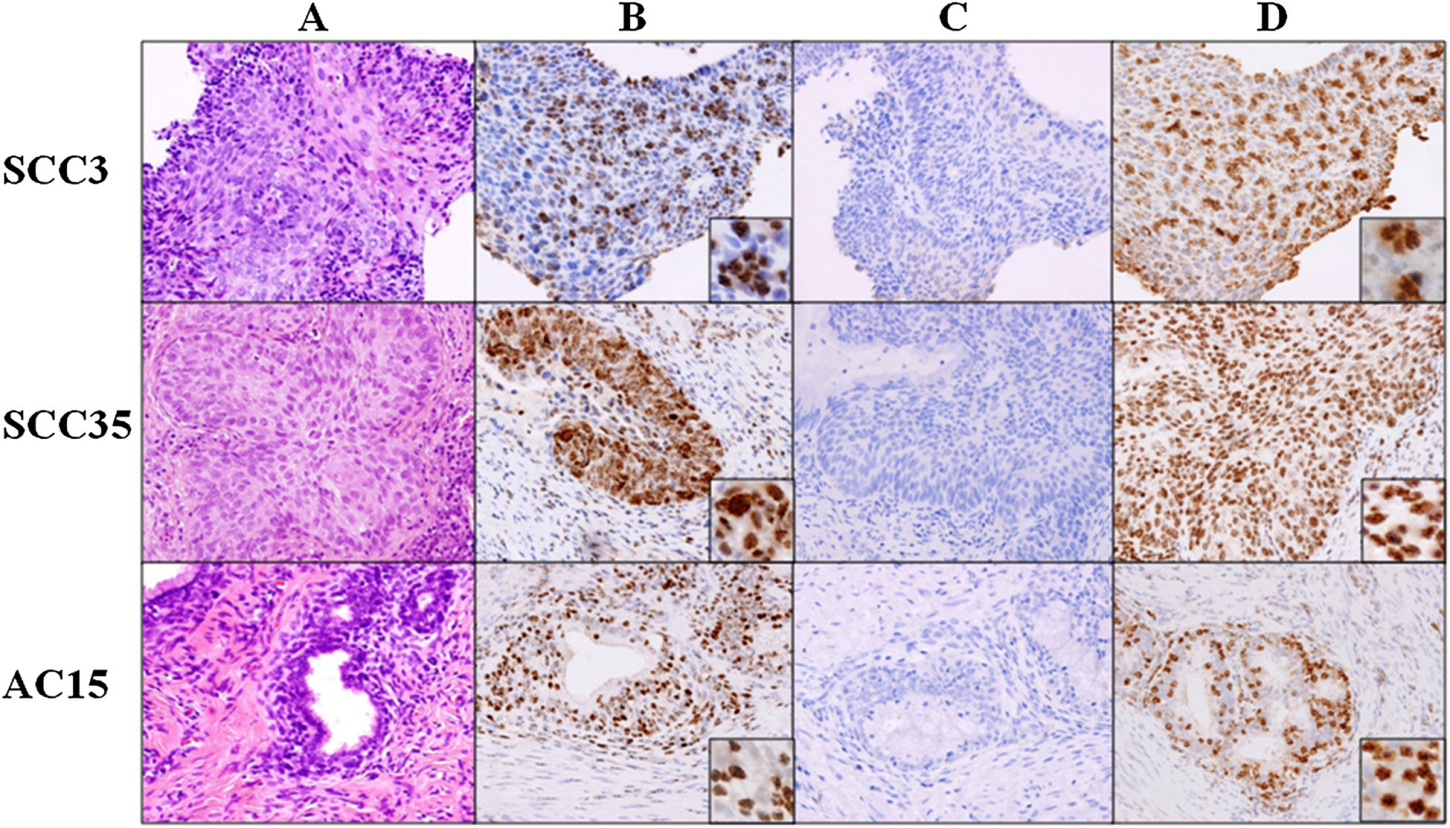
**Immunohistochemistry for detecting the MCPyV LT antigen on cervical SCC and AC tissue sections.** Hematoxylin and eosin staining (**A**) showed histological findings of the specimens containing SCC cells (cases SCC3 and SCC35) and AC cells (case AC15). Immunohistochemistry with the CM2B4 monoclonal antibody (**B**) and with the polyclonal antibody (**D**) showed immunoreactivity in the tumor cells. Insets show higher magnification views of the tumor cells. The isotype-matched negative control antibody (mouse IgG2b) for CM2B4 showed no immunoreactivity (**C**).

To evaluate the localization of MCPyV LT antigen expression, immunohistochemistry was performed using the mouse monoclonal antibody CM2B4 or a rabbit polyclonal antibody [23,39]. LT antigen is thought to be associated with oncogenesis of MCPyV-positive MCCs [40-43]. Semiquantitative immunohistochemistry findings in MCPyV PCR-positive cases are summarized in Table 1 and representative results are shown in Figure 3. Diffuse or speckled nuclear signals were observed in tumor cells, suggesting that these cancer cells harbored the MCPyV genome. Although in some cases weak staining was also observed in a small fraction of the adjacent non-neoplastic components including epithelial cells and lymphocytes, tumor cells had stronger nuclear immunoreactivity. Six MCPyV PCR-negative samples (three SCCs and three ACs) were also examined for the expression of LT antigen by immunochemistry, but no signals were detected (data not shown). Furthermore, the isotype-matched negative control antibody for CM2B4 showed no immunoreactivity either in cancer cells or in the surrounding normal components.

### Detection and typing of HPV DNA

The presence of the HPV *L1* gene was confirmed in all MCPyV-positive SCCs and ACs by PCR and by sequencing analysis for HPV typing (Table 1). Of nine MCPyV-positive SCCs, six cases were infected with HPV type 16, two with HPV 58 and one with HPV 31. HPV type 16 (two cases) and HPV 18 (two cases) were also found among the MCPyV-positive ACs.

## Discussion

MCPyV is thought to play a role in MCC tumorigenesis [1]. Although a causal link between MCPyV and other types of malignancy has not been established to date, recent studies have presented evidence of MCPyV detection in several cancers. Our previous findings showed that MCPyV was present in 4/30 cutaneous SCCs (13%) among Japanese patients [38]. A German group showed that 7/28 cutaneous SCCs (25%) were positive for MCPyV [35]. In other studies from North America, 26/177 cutaneous SCCs (15%) and 2/15 SCCs (13%) were positive for MCPyV [7,34]. Thus, the prevalence of MCPyV in cutaneous SCCs has been confirmed among distinct geographic populations. The present study demonstrated that the prevalence of MCPyV in cervical SCCs is close to that seen in cutaneous SCCs.

For detecting MCPyV, we used two primer sets targeting the *LT* and *VP1* regions, which gave different detection rates. Given the decreased amplification efficiency of larger amplicons by PCR of FFPE tissues, the LTsh primers should have detected MCPyV in more cases than the VP1 primers. Otherwise, PCR amplification might be hampered by mutations or deletions that exist in the primer regions, as suggested by recent studies [9,40].

The occurrence of false positive PCR results is unlikely. Our PCR runs were always performed using the appropriate controls and the negative controls were consistently negative in all experiments. To confirm that the PCR products contained MCPyV-specific DNA sequences but not artifacts, and to exclude the possibility of cross-contamination, we sequenced all the PCR products. Obvious variations in the DNA sequences were found in the MCPyV *VP1* gene. The sequencing results revealed the existence of three variants of the VP1 in our cases. The amino acid substitutions were present at three distinct positions, among which the replacement of glutamic acid with glutamine was found previously between two North American isolates, MCC350 and w162 [5]. Thus, amino acid substitutions are likely to occur frequently in MCPyV. The same was also reported among French MCPyV isolates [14]. On the other hand, amino acid substitutions at other locations would contribute to the antigenic diversity of the Japanese MCPyV. Any potential role of these substitutions remains to be elucidated.

We conducted immunohistochemistry of the MCPyV DNA-positive cervical SCCs and ACs to study the localization of MCPyV. CM2B4, a mouse monoclonal antibody to the MCPyV LT antigen, is available commercially and has been used widely for immunohistochemistry. Recently, a Japanese group generated a rabbit polyclonal antibody targeting a broader LT antigenic region than CM2B4 [39]. In addition to the CM2B4 monoclonal antibody, we employed this polyclonal antibody for immunohistochemistry in some cases. Both antibodies resulted in homogeneous or speckled nuclear staining of the tumor cells, indicating that MCPyV exists in cervical cancer cells. Nonspecific staining of the tissues is unlikely, because no signals were detected in the MCPyV PCR-negative samples and because our immunohistochemical method with the CM2B4 antibody was controlled by testing an isotype-matched control antibody. However, in some cases, weak immunoreactivity against these antibodies was also observed in a few surrounding normal cells. Therefore, we then performed PCR using DNAs extracted from normal tissues of the same patients with MCPyV PCR-positive cervical cancers, but neither the LTsh nor VP1 primers detected MCPyV DNA (data not shown). These findings suggest that the MCPyV genome was also present in nonneoplastic tissues of the uterine cervix at levels not detectable by PCR. The findings of semiquantitative immunohistochemistry did not correspond with the viral copy numbers detected by quantitative real-time PCR. A possible interpretation on the results would be that DNA in archived formalin-fixed tissues might be fragmented in the primer-targeting gene areas, and thereby the viral DNA copy numbers might be underestimated [44].

Currently, more than 100 HPV types have been identified and classified into high-risk and low-risk types according to the probability of developing a cervical cancer [45]. The high-risk HPV types are regarded as major causes of cervical cancer. Compared with Southeast Asia, Northern Africa, Europe and North America, HPV types 16 and 18 are less common and HPV types 31, 33, 52 and 58 are more common in Japanese patients with cervical cancers [46]. In the present study, the high-risk HPV types were detected predictably in all Japanese MCPyV-positive samples. These were HPV types 16, 31 and 58 in MCPyV-positive SCCs and HPV types 16 and 18 in MCPyV-positive ACs. The HPV typing pattern suggested no direct association of MCPyV with HPV types.

So far, research on the etiology of cervical cancers has focused on HPV. The present study provides the first evidence that MCPyV is present in a subset of HPV-associated cervical cancers. Because the MCPyV LT antigen is considered to be an oncoprotein responsible for the MCPyV-dependent oncogenic pathway [40-43], expression of the MCPyV LT antigen in HPV-associated cervical cancer cells suggests that MCPyV could be a cofactor of HPV for tumor initiation and/or progression. In the present study, an MCPyV-positive status had a tendency to be found at earlier clinical stages, although this needs further study. Recently, Houben et al. [47] presented new evidence that MCPyV might only be needed for tumor initiation, but additional mutations during tumor progression render LT antigen expression dispensable for MCC carcinogenesis. Although we do not know yet whether MCPyV is such a transient “hit and run” infectious pathogen or just a passenger during the development of cervical cancer, our findings should stimulate further investigations to clarify these important issues.

## Conclusions

This is the first observation presenting data on the prevalence of MCPyV in cervical cancers from Japanese patients. Our investigations indicate that MCPyV coexists in a subset of HPV-associated cervical cancers. Further worldwide epidemiological surveys are warranted to determine the pathogenetic relevance of MCPyV in cervical cancers.

## Methods

### Samples and DNA extraction

FFPE resection specimens were collected from the archives of Kochi University Hospital. The average ages of the patients with cervical SCCs and ACs were 43 years (range 28–74) and 54 years (range 41–80), respectively. According to the International Federation of Gynecology and Obstetrics (FIGO) clinical staging, the 48 SCC cases included 21 patients with stage 0, 7 patients with Ia, 2 patients with Ib, 2 patients with IIa, 3 patients with IIb, 1 patient with IIIa, 7 patients with IIIb and 5 patients with IVb. Of the 16 AC cases, there was 1 patient with stage 0, 9 patients with Ib, 3 patients with IIb, 2 patients with IIIb and 1 patient with IVb. All specimens were inspected by two independent pathologists and proved by histopathology to contain cancer cells. Two to five-μm thick sections were obtained from each FFPE tissue specimen and DNA was extracted using WaxFree DNA Kits (TrimGen Corporation, Sparks, MD, USA) according to the manufacturer’s instructions. This DNA was used as a template for the PCR analysis. In some cases, we also extracted DNA from non-neoplastic cervical FFPE sections from the same patients with cervical cancers. This study was approved by the Ethics Committee of Kochi Medical School, Kochi University (approval number 2224).

### Identification of MCPyV and HPV by PCR analysis

PCR was conducted with 200 ng of extracted DNA using an AmpliTaq Gold 360 master mix (Life Technologies, Tokyo, Japan) and 0.4 μM of each primer in a total volume of 50 μL. For detecting MCPyV DNA, two primer sets designated LTsh and VP1 were employed (Table 2). The LTsh primers were designed to detect a region of exon 2 in the MCPyV *LT* gene. The forward and reverse primers are located at nucleotide positions 910–930 and 1133–1152, respectively, based on the GenBank sequence EU375803. The VP1 primers target the MCPyV *VP1* gene region [1]. The GP5^+^/6^+^ primer set was used to detect HPV DNA [48]. The β-globin gene was also amplified in separate PCR runs as a positive control to confirm the presence of PCR-amplifiable DNA. As a PCR-negative control, water containing all PCR components except for DNA was used. The reaction mixtures were denatured at 95°C for 10 min and then amplified with 40 cycles at 95°C for 30 s followed by 55°C for 30 s for MCPyV, or 45°C for 30 s for HPV and 72°C for 30 s for MCPyV, or 72°C for 10 s for HPV, with a final extension of 7 min at 72°C. The PCR products were electrophoresed on 2.5% agarose gels, visualized with ethidium bromide staining and photographed using the Kodak EDAS 290 gel documentation system (Kodak, Rochester, NY, USA).

**Table 2:** Primer sequences used in this study

**PCR analysis and DNA sequencing analysis**			
**Target gene**	**Primer name**	**Sequence (5’ → 3’)**	**Predicted product size**
MCPyV *LT*	LTsh-F	GATCAGGAGGATTCAGCTTCG	242 bp
	LTsh-R	CAGAGGATGAGGTGGGTTCC	
MCPyV *VP1*	VP1-F	TTTGCCAGCTTACAGTGTGG	352 bp
	VP1-R	TGGATCTAGGCCCTGATTTTT	
HPV *L1*	GP5+	TTTGTTACTGTGGTAGATACTAC	150 bp
	GP6+	GAAAAATAAACTGTAAATCATATTC	
Human β-globin	β-globin-F	ACACAACTGTGTTCACTAGC	110 bp
	β-globin-R	CAACTTCATCCACGTTCACC	
**Real-time PCR analysis**			
**Target gene**	**Primer sequence (5’ → 3’)**	**Probe sequence (5’ → 3’)**	
MCPyV *ST*	GCAAAAAAACTGTCTGACGTGG	FAM-TATCAGTGCTTTATTCTTTGGTTTGGATTTCCTCCT-TAMRA	
	CCACCAGTCAAAACTTTCCCA		
Human RNase P	AGATTTGGACCTGCGAGCG	FAM-TTCTGACCTGAAGGCTCTGCGCG-TAMRA	
	GAGCGGCTGTCTCCACAAGT		

### DNA sequencing analysis

The PCR products were purified with High Pure PCR Product Purification Kits (Roche Diagnostics, Tokyo, Japan) and then sequenced directly with ABI Big Dye Terminator 1.1 Cycle Sequencing Kits (Life Technologies). The sequenced products were analyzed using a model 3130 genetic system (Applied Biosystems, Tokyo, Japan). For identifying HPV types, the obtained sequencing data were BLAST searched using the National Center for Biotechnology Information (NCBI) database (http://www.ncbi.nlm.nih.gov/blast/Blast.cgi). The MCPyV *LT* and *VP1* sequencing data were aligned using the BioEdit program and then compared with reference sequences of the North American MCPyV isolate, MCC350, and the Japanese MCPyV isolate, TKS (GenBank accession numbers EU375803 and FJ464337, respectively).

### Quantitative real-time PCR

Quantitative real-time PCR was conducted with 500 ng of extracted DNA, according to the method of Bhatia et al. [49] with some modifications. The primer and probe sequences are shown in Table 2. Standard PCR was conducted using the same primers and the PCR product was cloned into the pMD20-T vector (TaKaRa, Shiga, Japan). We prepared six-fold serial dilutions using 10 ng of the cloned plasmid DNA to generate a standard curve and we calculated the copy number in each sample. The viral DNA load was defined as viral DNA copies per RNase P gene copy, which represented the copy number per cell.

### Nucleotide sequence accession numbers

The obtained MCPyV *LT* and *VP1* sequences were deposited in the NCBI database under accession numbers [AB645848, AB645849, AB645850, AB645851, AB645852, AB645853, AB645854, AB645855, AB645856, AB645857, AB645858, AB645859, AB645860, and AB645861].

### Immunohistochemistry

For detecting MCPyV LT antigen expression, immunohistochemistry was performed on FFPE tissue sections using a mouse monoclonal antibody CM2B4 (IgG2b isotype) [23] or a rabbit polyclonal antibody [39]. Samples sectioned at 4 μm were deparaffinized and rehydrated. Heat-induced epitope retrieval was performed using EDTA antigen retrieval buffer for 30 min (Dako, Tokyo, Japan). Endogenous peroxidase activity was blocked using 3% hydrogen peroxide in water for 10 min. Following incubation with blocking solution for 10 min, slides were incubated with the CM2B4 antibody diluted with 0.05 mol/L Tris–HCl buffer containing 0.1% Tween 20 at 1:100 or with the rabbit polyclonal antibody at a dilution of 1:2000 for 20 min at room temperature. After sufficient washes in 0.05 mol/L Tris–HCl buffer solution containing 0.3 mol/L NaCl, and 0.1% Tween 20 (TBST), horseradish peroxidase-conjugated goat anti-mouse or anti-rabbit immunoglobulin was applied as the secondary antibody for 15 min at room temperature. After further washes in TBST, bindings of the primary antibodies were detected using a biotin-free tyramide signal amplification system (Dako) according to the manufacturer’s instructions. Finally, sections were counterstained with hematoxylin. The specificity of staining with CM2B4 was controlled by testing an isotype-matched control mouse IgG2b (Dako) in parallel.

Immunohistochemistry for detecting CK20 and chromogranin was performed as described [22]. For all immunohistochemical markers, cases were scored as – (negative), 1+ (<10% cells immunoreactive), 2+ (10–50% cells immunoreactive), or 3+ (>50% cells immunoreactive).

## Competing interests

The authors declare that they have no competing interests.

## Authors’ contributions

MI carried out the PCR analysis and DNA sequencing, analyzed the data and drafted the manuscript. YH carried out the PCR analysis and DNA sequencing. YN collected clinical samples and conducted the immunohistochemistry. HO, NM and TF provided the clinical samples and clinical data. MF participated in the immunohistochemistry. MD conceived the study, conducted immunohistochemistry, contributed to the acquisition of funding and revised the manuscript. All authors read and approved the final manuscript.

## Author details

^1^Department of Microbiology and Infection, Kochi Medical School, Kochi University, Nankoku, Kochi 783–8505, Japan. ^2^Department of Obstetrics and Gynecology, Kochi Medical School, Kochi University, Nankoku, Kochi 783–8505, Japan. ^3^Department of Pathology, Kochi Medical School, Kochi University, Nankoku, Kochi 783–8505, Japan.
